# Waterdock 2.0: Water placement prediction for *Holo*-structures with a pymol plugin

**DOI:** 10.1371/journal.pone.0172743

**Published:** 2017-02-24

**Authors:** Akshay Sridhar, Gregory A. Ross, Philip C. Biggin

**Affiliations:** Department of Biochemistry, University of Oxford, Oxford, United Kingdom; University of Minnesota Twin Cities, UNITED STATES

## Abstract

Water is often found to mediate interactions between a ligand and a protein. It can play a significant role in orientating the ligand within a binding pocket and contribute to the free energy of binding. It would thus be extremely useful to be able to accurately predict the position and orientation of water molecules within a binding pocket. Recently, we developed the WaterDock protocol that was able to predict 97% of the water molecules in a test set. However, this approach generated false positives at a rate of over 20% in most cases and whilst this might be acceptable for some applications, in high throughput scenarios this is not desirable. Here we tackle this problem via the inclusion of knowledge regarding the solvation structure of ligand functional groups. We call this new protocol WaterDock2 and demonstrate that this protocol maintains a similar true positive rate to the original implementation but is capable of reducing the false-positive rate by over 50%. To improve the usability of the method, we have also developed a plugin for the popular graphics program PyMOL. The plugin also contains an implementation of the original WaterDock.

## Introduction

Protein-ligand interactions are fundamental to many cellular processes and understanding them is crucial for adopting a rationalized approach to drug-design. Water molecules, with their ability to form multiple bridging hydrogen bonds, have been identified as a key structural factor in mediating these interactions [1–8]. In cases such as the L-arabinose binding protein, the water molecules are a pharmacophoric feature of the binding site and allow discrimination between ligands [9]. Conversely, in other cases—like the oligopeptide binding protein (OppA)—water molecules promote promiscuity by acting as flexible adapters, facilitating a range of ligands to bind [10]. Water mediated interactions are so ubiquitous that a comprehensive analysis of 392 high-resolution crystal structures found 85% of the protein-ligand interfaces having at-least one ‘bridging’ water molecule [11].

With their importance and prevalence, water is increasingly being included in a variety of computational binding studies [12–19]. In quantitative structure-activity relationship (QSAR) modelling, Hussain *et al*. [20] found that the incorporation of explicit water molecules in the binding-site of actin enabled improved accuracy for ligands with the formamide moiety. Similarly, Taha et al. [21] incorporated water molecules in their 3D contact analysis search for inhibitors of candida N-myristoyl transferase (NMT) and glycogen phosphorylase (GP). A number of groups have also used crystallographic water molecules in molecular docking screens. Huang et al. [22] used explicit water sites in 24 proteins to improve their docking enrichment factors and reduce false positives. Verdonk et al. [23] also used crystallographic waters to improve docking performance by up to 20%. The use of water molecules in docking studies has become so ubiquitous that programs like AutoDock4 [24], Gold [23], Rosetta [25], Glide [26] and FlexX [27] all offer options to include explicit waters.

However, utilising water molecules in binding studies first necessitates an accurate knowledge of their locations. Water positions are typically obtained from high-resolution crystallographic structures. However, in many instances, the protein structure is often obtained via methods such as NMR or homology modelling that do not provide this information. For such cases, peaks in water density derived from Molecular Dynamics (MD) or Monte-Carlo (MC) simulations with explicit water can suggest likely water positions [28]. However, the method can have long convergence timescales (up to hundreds of microseconds) for buried binding sites due to the time it takes for explicit water molecules to permeate within the protein [29]. Moreover, the use of water molecules in large-scale molecular screening requires an expeditious prediction of their locations. Hence, various ‘fast-solvation’ methods have been developed to swiftly estimate the locations of water within protein structures [30–37].

WaterDock [38] is one such algorithm that uses the freely available AutoDock Vina [39] tool to predict the water locations within the binding pocket. Water molecules are initially treated as ligands and are docked thrice into a binding site of the protein. With AutoDock Vina able to predict up to 20 configurations per run, the initial docking results in a maximum of 60 probable water co-ordinates. A Vina score cut-off of ≤ − 0.6 kcal/mol is then applied to the co-ordinates to remove water-sites that are energetically unfavourable. The ensemble of water co-ordinates is then sequentially clustered twice using the single linkage method with distance cut-offs of 0.5 Å and 1.6 Å. WaterDock was able to predict 88% of the water-sites in a dataset of seven high-resolution crystal structures. When validated against a set of 14 OppA crystal structures used by the AcquaAlta method [40], WaterDock could predict 97% of the water-sites.

However, in tests against consensus water-sites from seven crystal structures, WaterDock had a false-positive rate of 24% [38]. In the OppA dataset, the false positive rate was on average 1–2 waters per structure. Whilst this rate of false-positive prediction may be tolerable for some applications, it may pose problems for large-scale automated workflows. Therefore, we were keen to see if this aspect of the WaterDock method could be improved. Recent work on the solvation structure of small molecules has indicated a correlation between their hydration shells and the location of mediating waters in *holo*-structures [41,42]. In this work, we show how information from hydration shells can be combined with the WaterDock protocol to give an improved false-positive hit rate and incorporate the entire workflow in to an easy to use PyMOL [43] plugin.

## Methods

### Overview of the WaterDock 2.0 pipeline

**Fig 1:**
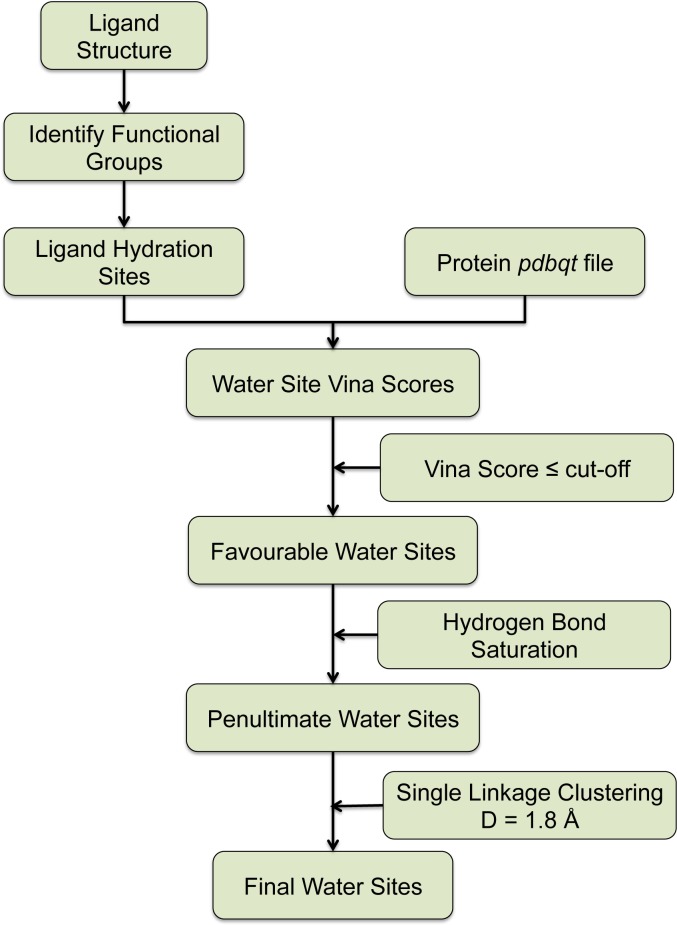
Schematic illustration of the WaterDock 2.0 bridging water prediction pipeline.

The pipeline of WaterDock 2.0 for identifying bridging water molecules in *holo* structures is outlined in **Fig 1**. In the original WaterDock protocol, the position of the waters within the structure were predicted without consideration of the hydration and hydrogen bonding capability of the ligand. To address this we analysed the solvation behaviour of small molecules from MD simulations.

Functional groups of the ligand are first identified and their hydration structures are generated semi-empirically as detailed below. Sites within this hydration structure with ‘favourable’ protein-water interactions are then identified using AutoDock Vina. To do this, a water molecule is iteratively docked onto the protein with a small box-size of 0.5 Å centred on coordinates from the ligand hydration shell. From AutoDock Vina version > 1.0.2, the algorithm is adjusted to not exclude results whose hydrogen atoms are outside the search space. Thus, the small box size allows the usage of Vina’s docking function to score the site’s water-protein interaction by preventing its lateral displacement. The ‘*num_modes*’ option of Vina that controls the number of output configurations was set to 1 to allow generation of only the most favourable configuration. Additionally, the small box size allows the ‘*exhaustiveness*’ option to be set to 5 (compared to 20 in the original protocol).

Subsequently, and as described for the original WaterDock, unfavourable water-sites with a Vina score more positive than an empirically calculated cut-off (in this case -0.55 kcal/mol) are discarded.

Our strategy to reduce the number of false-positive results is to employ a hydrogen bond saturation limit. Essentially, this dictates the maximum number of bridging water-sites from the hydration shell of each functional group. Thus, if more than the stipulated number of water-sites from a motif’s hydration shell are predicted to have favourable Vina scores, only the highest scoring ones among them are selected. The saturation limits were selected based on the number of valence shell electron pair repulsion (VSEPR) lone pairs or bound hydrogens in the oxygen/nitrogen atoms. **Table 1** lists the functional groups implemented in WaterDock 2.0 and the corresponding H-bond saturation limit. Finally, water-sites where the centres of the waters are within 1.8 Å of each other are clustered to avoid the problem whereby hydration sites subtended by multiple ligand functional groups are predicted as multiple bridging waters.

**Table 1:** The H-bond saturation limit enforced on the various functional groups implemented in the WaterDock 2.0 pipeline.

**Motif**	**H-bond Limit**
Carbonyl	2
Carboxyl	2
Cyano	1
Imine	1
Nitro	2
Amine	No. of H
Sulfonyl	2
Phosphoryl	2
Hydroxyl	3
Ether	2
Halogen	1

### Identifying functional group hydration

#### Dataset

To semi-empirically generate the hydration shells of ligands, the individual hydration of functional groups was first calculated from Molecular Dynamics (MD) simulations of ligands from the CSAR-2012 dataset [44]. The Community Structure-Activity Resource (CSAR) is a regularly compiled dataset of crystal structures aimed to provide benchmarks for the development of scoring functions and docking algorithms. The first set of the 2012 database contains 242 high-resolution crystallographic structures with no regions of ambiguously resolved ligand electron density. With the inherent inaccuracies associated with the identifying water molecules from crystallography, the dataset has limited use in the development of prediction algorithms. However, the structural accuracy and the large number of molecules in the dataset make it ideal for compiling the hydration of specific functional groups across a range of chemical environments.

#### Methodology

Ligand structures were visually inspected for their correct protonation states before parameterisation according to the General Amber Force Field (GAFF) [45] using Antechamber [46] and Amber 14 [47]. The partial charges were calculated according to the AM1-BCC method [48,49] and the parameters were converted to GROMACS format using the *acpype* script [50]. The ligands were then solvated in a TIP3P rectangular box of water with a minimum distance of 14 Å from each edge and neutralised by the addition of Na^+^/Cl^−^ ions as parameterised by Joung et al. [51]. GROMACS 5.0.2 [52] was used to simulate all boxes for 30 ns each with a time-step of 2 fs. The temperature was maintained at 300 K and the pressure at 1 bar using the V-rescale thermostat and the Parrinello-Rahman barostat [53] respectively. Co-ordinates were saved every 0.6 ps resulting in 50000 trajectory frames. During the course of the simulation, the conformation of the ligands were maintained using positional restraints of 100 *kJ/mol nm*^*2*^ on all non-hydrogen ligand atoms.

The hydration shells of ligands were discretised using the Quality Threshold (QT) algorithm [54] similar to the methodology of *WATSite* [55] and *Placevent* [56]. First, a 3-D grid with a spacing of 0.25 Å and extending up to 4 Å from all heavy atoms was placed over the ligand. Subsequently, the residence of the oxygen atom of water in each grid-point was tabulated across all frames of the trajectory. This tabulated grid is then histogrammed to provide a 3-D occupancy density matrix. Finally, the QT algorithm is used to discretise the histogram with a minimum distance of 2 Å between clusters.

For each ligand, discretised hydration sites were ‘assigned’ to the nearest functional group and the ‘assigned’ hydration sites of each motif were then overlaid across all ligands of the dataset. This allowed us to make a comprehensive picture of probable functional group hydration distribution across different atomic surroundings.

### Training

#### Dataset

The Vina score cut-off for the prediction algorithm was calculated using the Astex diverse dataset [57] of 85 *holo* structures. Compiled from the Protein Data Bank [58], the ligands are structurally diverse and, more importantly, the resolved electron density accounts for all parts of the ligand. While not all relevant bridging water molecules are resolved within the dataset, studies by Hartshorn et al. [57] showed that the resolved waters are accurate and capable of improving docking performance by up to 20%. Thus, this validation set was chosen to find the energetic cut-off score that allows the prediction of the maximum number of water-sites. However, with not all bridging waters resolved in the crystallographic structures, a fairly high false-positive rate was anticipated in this training set.

#### Methodology

Hydrogen atoms were added to the ligand structures of the Astex dataset using the Reduce program [59] and their protonation states were visually verified. The polar motifs of the molecule were then used to ‘model’ the hydration shell of the molecule as per the results from the previously simulated CSAR-2012 dataset. The protein-water interactions for each of the modelled hydration sites was scored using AutoDock Vina through iterative docking as described above. Finally, for each of the modelled water-sites, the distance to the nearest crystallographic water was calculated and considered ‘conserved’ if the distance was less than 2.0 Å.

#### Validation

The co-ordinates of water molecules are notoriously difficult to accurately estimate [60]. During the initial development of WaterDock [38], this was circumvented by overlaying multiple independently crystallised structures and only considering those sites resolved at least twice. This overlaying allows the accounting for variations in crystallographic conditions and errors during the refinement process [61]. A similar dataset was compiled to validate this new WaterDock 2.0 workflow. **Table 2** lists the details of the validation dataset and the number of consensus water-sites in each case. The ligands in each structure were overlaid and the water-coordinates within 3.2 Å of both ligand and protein present in more than one structure were considered as consensus sites. This may seem a generous cut-off, but we wanted to be certain that all relevant waters were included. Additionally, to allow a direct comparison with the original WaterDock and AcquaAlta algorithms, the 14 OppA structures used by both were also analysed.

**Table 2:** The dataset used to validate the ligand-directed Waterdock 2.0 algorithm.

**Protein**	**PDB Codes**	**Ligand Code**	**Resolution (Å)**	**Consensus Waters**
HIV-1	3FXS, 2ZYE	KNI-272	0.93, 1.9	8
GluR2	1FTM^a^, 1MY2^a^	AMPA	1.7, 1.8	18
Trypsin	2AH4, 3RJX	GBS	1.13, 1.7	8
GST	1K3Y^a^, 1K3L^a^	GTX	1.3, 1.5	22
HSP90	2BRC, 2BT0^a^	CT5	1.6, 1.9	6
PIM1	1XWS, 2BIK	RBT205	1.8, 1.8	4
Bromodomain	3ZYU^a^, 4ALG	I-BET	1.5, 1.6	6
Androgen Receptor	4OHA, 2AX6	Hydroxyflutamide	1.42, 1.5	4
Casein Kinase II	3BQC, 3Q9W	Emodin	1.5, 1.7	4
Thrombin	4CH2^a^, 5LUW	0G6	1.6, 1.69	14
Carbonic Anhydrase	3HS4, 3V2M, 3DC3	Acetazolamide	1.1, 1.47, 1.7	3

^a^—Structures where multiple chains were overlaid to validated waters.

## Results and discussion

### Functional group hydration

**Fig 2:**
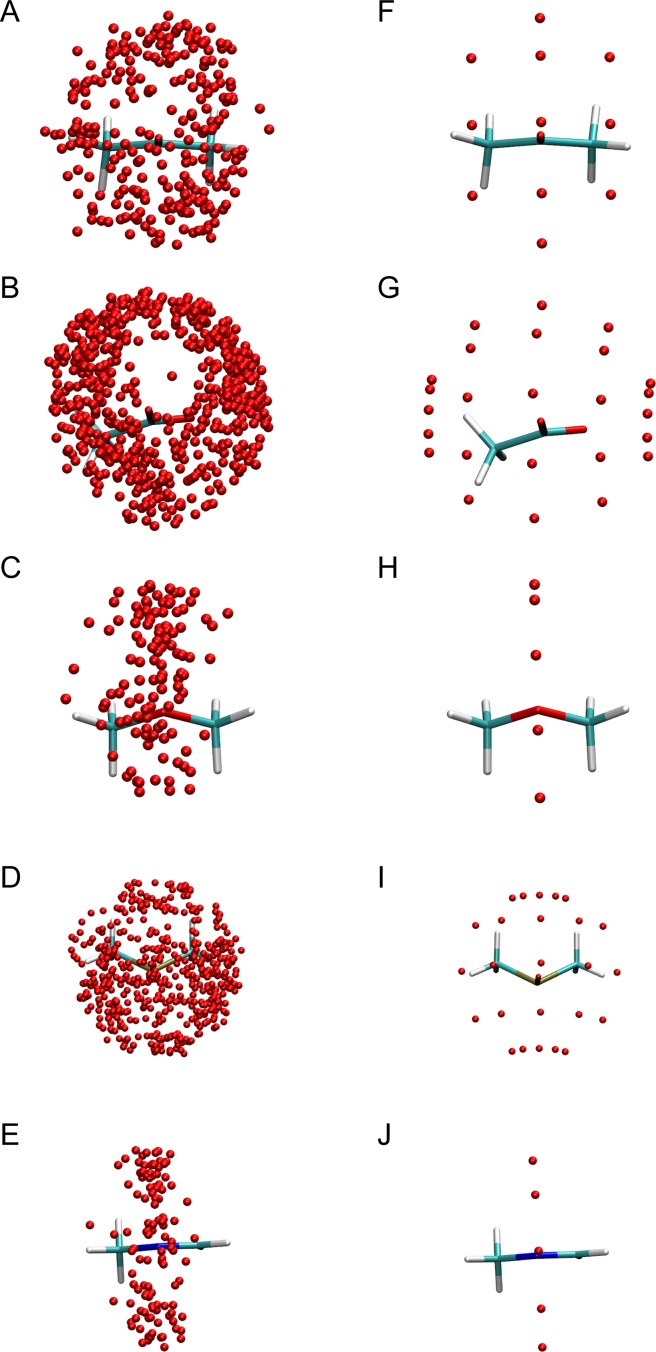
The MD (**A-E**) and modelled (**F-J**) hydration structure of five polar functional groups. **(A, F)** carbonyl, **(B, G)** carboxyl, **(C, H)** ether, **(D, I)** phosphoryl, **(E, J)** imine.

The total hydration structure calculated from MD around five polar functional groups (carbonyl, carboxyl, ether, phosphoryl and imine) are shown in **Fig 2A–2E**.

The orientations of water-sites around each group are further analysed as a distribution of φ and θ - the angles made by the ligand atom—O_water_ vector with an imaginary **X** and **Z** axes (see **S1 Fig**). Hydration sites around carbonyl oxygens are distributed in a uniform ‘oval’ shape with θ ranging from 40° to 140° and φ ranging from 20° to 160°. The distribution around carboxyl oxygens is also ‘oval’ albeit with a larger range of angles with θ spanning from 60° to 160° and φ spanning from 0° to 180°. This increased range of binding might probably be attributed to a combination of the larger solvent accessibility of carboxyl oxygens and the greater negative charge on the oxygen atoms. Additionally, θ deviates from a mean value of 90° seen in carbonyl oxygens to **∼**110° probably due to hydration sites also interacting with the other oxygen atom of the carboxylic motif. In phosphoryl oxygens, the distribution of hydration sites is similar to that seen in carboxylic acids with a mean θ value again deviating from 90°. This deviation might probably be caused by a similar reason with phosphoryl oxygen atoms normally occurring as pairs in ligand molecules. The hydration sites of both ether oxygen and imine nitrogen lie on a plane normal to that of the motif with θ not deviating from **∼**90°.

**Fig 3:**
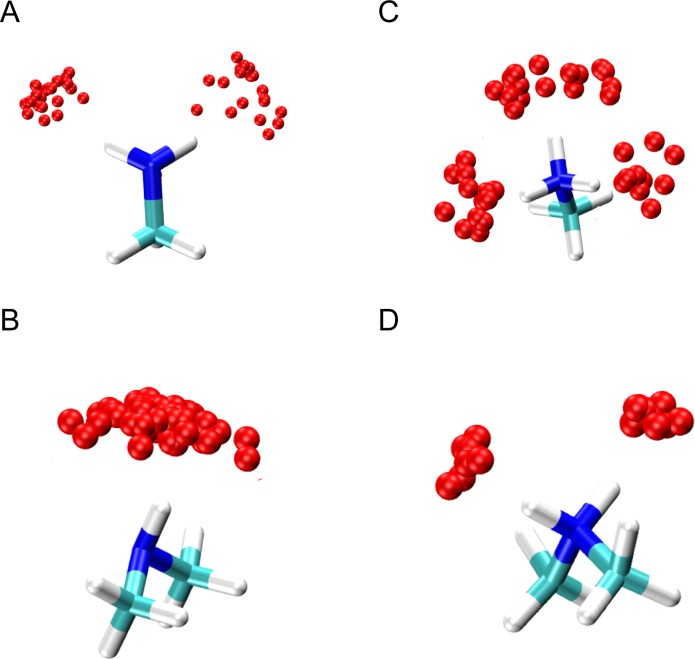
The location of water-sites around different amine groups as produced in MD.

**Fig 2F–2J** show the modelled hydration structures of the five functional groups that have been fitted to the simulation results. The polar atom—O_water_ vector length was modelled with a length 3.0 Å to match the experimental hydrogen bond length [62] and distinct water-sites were modelled 2.0 Å apart. **Fig 3** shows the location of water-sites derived from MD simulation around different primary, secondary and tertiary amines. In all cases, water-sites preferentially bind to the hydrogen atom. This preference for linearity in the N-H—O_w_ binding is similar to those observed in Neutron Diffraction [63,64] and CSD mining studies [40]. Hence, amine hydration sites are modelled along the direction of the N-H vector with a distance of 3 Å from the nitrogen atom.

**Fig 4:**
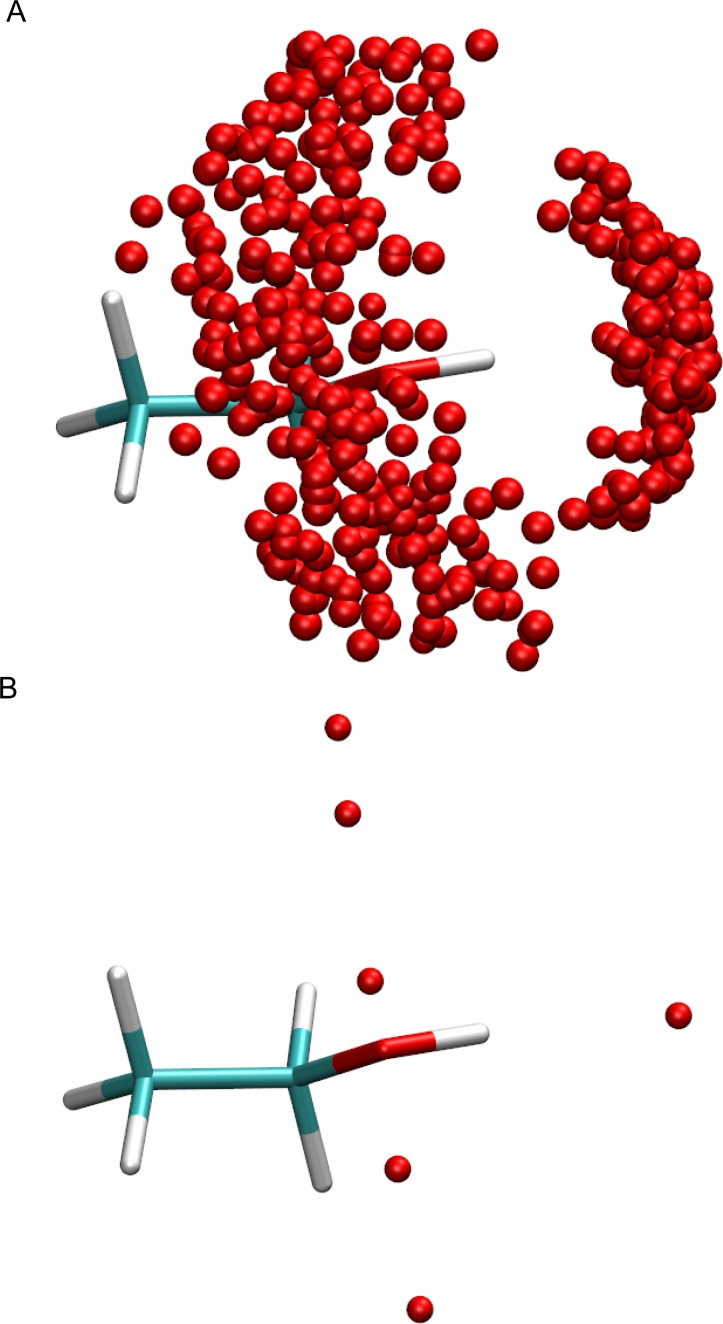
**(A)** The distribution of hydration sites around the hydroxyl functional group from MD simulation. **(B)** The modelled hydration structure of the hydroxyl functional group.

**Fig 4A** shows the hydration structure of hydroxyl groups from simulation. Two distinct orientations of water are seen bound to the hydrogen and oxygen atoms. Therefore, the hydration of the hydroxyl group is modelled as a combination of an ‘ether’ oxygen atom and an ‘amine’ hydrogen atom (see **Fig 4B**).

Of the 55 chloride functional groups in the dataset, none had a water-site distributed around it in MD simulation. On the other hand, fluorine atoms were hydrated. However, with only four F atoms in the dataset, an accurate hydration distribution could not be determined. Similarly, insufficient ligands with ‘nitro’, ‘sulphonyl’ and ‘cyano’ functional groups were present in the dataset. The hydration of all four functional groups were thus modelled similar to that of the most extensively hydrated motif—carboxyl oxygen Empirical knowledge of the hydration of these functional groups can plausibly be gathered from the Cambridge Structural Database [65,66] using SuperStar [67]. However, the high prediction accuracy of the current modelling method (see below) did not necessitate the usage of proprietary software.

### Establishing the Vina score cut-off

**Fig 5:**
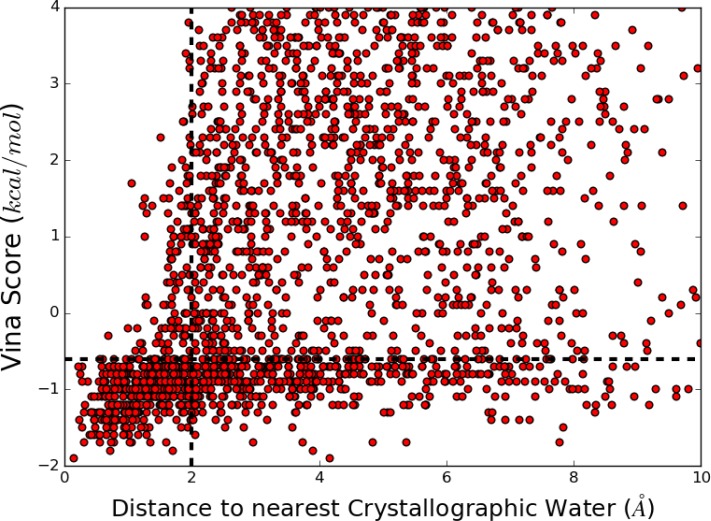
Scatter plot showing the water-site’s Vina docking score against the distance to the nearest crystallographic water on application of the WaterDock 2.0 pipeline to the Astex Diverse Set. The 2.0 Å distance cut-off is plotted as a vertical dotted line and the Vina cut-off score of -0.55 kcal/mol is plotted as a horizontal dotted line. The lower left quadrant thus signifies crystallographic waters molecules correctly identified within the training set.

Modelling the hydration sites based on functional groups predicted 2045 water-sites around the 85 ligands of the Astex diverse Set [57]. **Fig 5** plots the results of the docking study on each of the 2045 semi-empirically generated water-sites. It shows a scatter plot of the site’s docking Vina score against the distance to the nearest crystallographic water. The 2 Å distance cut-off used in the original WaterDock protocol for discriminating ‘conserved’ and ‘displaced’ water molecules is also plotted as a dotted vertical line. The Kendall rank correlation coefficient between the Vina score and distance to a crystallographic water site was found to be 0.29 with a p-value less than or equal to 2.2×10^−16^. Therefore, there is a weak, but very significant association between the Vina score and accuracy, such that ligand hydration sites with stronger binding scores are more likely to be ‘conserved’ and vice-versa.

Using the statistical program **R** with the *rpart* package, a regression tree with a single split was used to calculate a cut-off score with which to discard the most unfavorable possible water locations. The cut-off that was most able to identify (using the Gini index) predictions that were no greater than 2 Å from crystallographic water sites was found to be -0.55 *kcal/mol* (shown as black horizontal line in **Fig 5**). Thus, all hydration sites with a Vina score more negative than -0.55 *kcal/mol* are retained for hydration bond saturation analysis and clustering.

### Validation of the protocol

**Fig 6:**
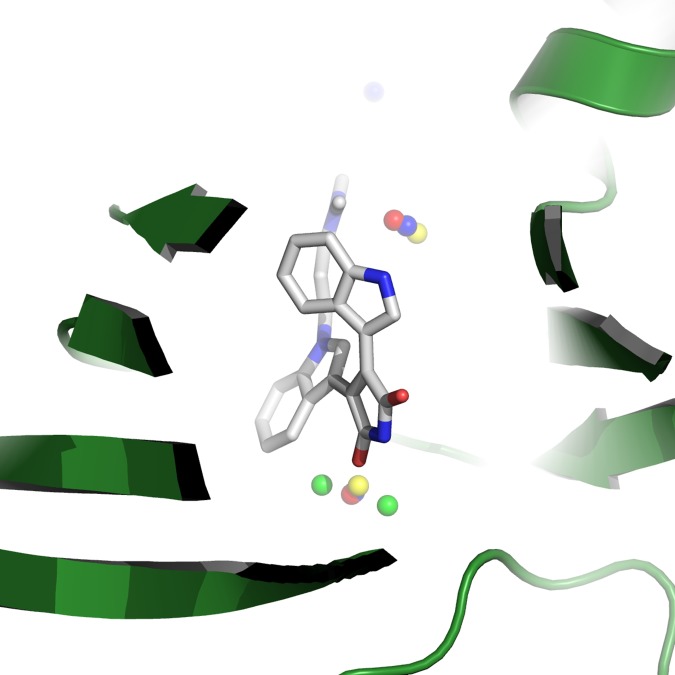
The location of the crystallographic waters from two structures of PIM-1 with PDB accession codes 1XWS and 2BIK, where the crystallographic waters from the two structures are shown in red/blue and the predicted waters from the two runs are shown in green/yellow, respectively. A false-positive result arises from two sites (green) predicted adjacent to the same crystallographic water (red/blue).

**Table 3** lists the results of the WaterDock 2.0 pipeline on the validation dataset of *holo* structures. A water-site was considered correctly predicted if it was within 2.0 Å of both water molecules used to identify a consensus site. The protocol has an identical true positive rate to the original WaterDock of 88%. However, the incorporation of ligand conformation and hydration reduced the false-positive rate from ~24% to ~8%. Even with this improvement it is still of interest to investigate cases where the method struggles. **Fig 6** shows the predicted and crystallographic water-sites from the two PIM-1 structures 1XWS and 2BIK. The crystallographic waters from the two structures are shown in red/blue and the predicted waters are in green/yellow. The false-positives in this structure are a result of a ‘chaining’ of predicted of waters with two sites predicted either site of the same water molecule. Thus, despite the double-predictions inflating the number of false-positives, the results are still within hydrophilic regions of the *holo-*structure.

**Table 3:** The results of the validation dataset of eleven protein holo-structures.

**Protein**	**Consensus Waters**	**Total Predicted Waters**	**Predicted Consensus Waters**	**False-Positives**
HIV-1	8	8	8	0
GluR2	18	18	18	0
Trypsin	8	5	5	0
GST	22	22	18	4
HSP90	6	7	6	1
PIM1	4	5	4	1
Bromodomain	6	6	6	0
Androgen Receptor	4	4	4	0
CK II	4	5	3	2
Thrombin	14	13	11	2
Carbonic Anhydrase	3	3	3	0
TOTAL	97	96	86	10

Additionally, the new pipeline proposed here sacrifices AutoDock Vina’s search algorithm for semi-empirically generated probable sites. This results in a slightly greater mean error of 1.24 Å compared to 0.83 Å in the original WaterDock protocol.

**Table 4** lists the results of the OppA dataset used to validate the protocol and offer a further comparison to WaterDock and AcquaAlta. AcquaAlta was validated using a cut-of distance of 1.4 Å and could predict 66% of the 103 crystallographic waters. At the same maximum error, the original WaterDock could predict 87% of bridging waters. The slightly greater maximum error of the new protocol becomes evident in the OppA dataset with WaterDock 2.0 able to predict only 78 water molecules (76%) at 1.4 Å. While inferior to the original protocol at this cut-of distance, the new pipeline still out-performs AcquaAlta. However, if the maximum error was increased to match the modelled inter hydration-site distance of 2.0 Å, the prediction rate increases to 91%. Only 8 false-positive sites were predicted compared to 19 using the original protocol. Using the **R** package *exact*, Boschloo’s exact test was used to determine the statistical significance of this improvement, given a fixed number of predictions for the original WaterDock and WaterDock 2.0. Under the null hypothesis that WaterDock and WaterDock 2.0 have the same false positive rate, the probability for observing 8 false positives or fewer with WaterDock2 (i.e. the p-value) was calculated to be 0.033, which is significant up to standard 0.05 level. The number of true positives for WaterDock 2.0 was 92, compared to 95 true positives of the original WaterDock. Using Boschloo’s exact test for a fixed total number of predictions, the null hypothesis of equal true positive rates was not rejected with a p-value of 0.979. A further comparison between the two WaterDock protocols is provided in S2 Fig.

**Table 4:** The results of the OppA dataset of structures used to allow comparison of new prediction protocol to AcquaAlta and the original WaterDock methodologies.

**Structure PDB code**	**Waters**	**Predictions (1.4 Å)**	**Predictions (2.0 Å)**	**False Positives**
1JET	7	5	6	0
1JEU	9	7	8	1
1JEV	6	5	5	0
1B4Z	10	7	9	1
1B5I	7	5	7	1
1B32	7	5	6	0
1B3F	7	5	6	1
1B46	6	4	6	0
1B51	9	7	8	0
1B58	7	5	6	0
1B5J	10	8	8	1
1B9J	6	5	6	1
1QKA	6	6	6	1
1QKB	6	4	5	1
Total	103	78	92	8

## Conclusions

To summarise, the inclusion of ligand conformation and its associated hydration sites into the WaterDock pipeline allows accurate prediction of bridging waters. Additionally, a consideration of the conformation of the ligand polar groups allows a marked reduction in the number of false-positives compared to the original WaterDock protocol. Finally, the semi-empirical generation of hydration sites allows a robust application to *holo* structures of different ligand sizes without needing to consider effects of changing box sizes (the clustering in the original WaterDock implementation was tuned for a cube of sides 15 Å and thus very large ligands may prove problematic as recently discussed in similar applications [31]).

WaterDock 2.0 was envisaged with a view of combining ligand hydration with AutoDock Vina’s scoring to predict water molecules within the binding site of *holo* structures. While the validation of any prediction protocol is inherently difficult considering the inaccuracies associated with crystallographic waters, the new pipeline significantly reduced the number of false-positives while matching the true positive rate of WaterDock. While the speed of predictions is reduced due to the greater number of docking attempts in the new protocol, the effect is marginal due to the significant reduction in the ‘*exhaustiveness*’ and search space of each run.

The speed of the protocol and its sensitivity to ligand conformation (because final water positions are generated based on the orientation of the functional groups) make it ideal for combination with drug screens. Work is currently underway to combine WaterDock 2.0 with docking poses to predict conformation-dependant bridging waters which in-turn can be used to develop a ‘hydrated’ docking score.

**Fig 7:**
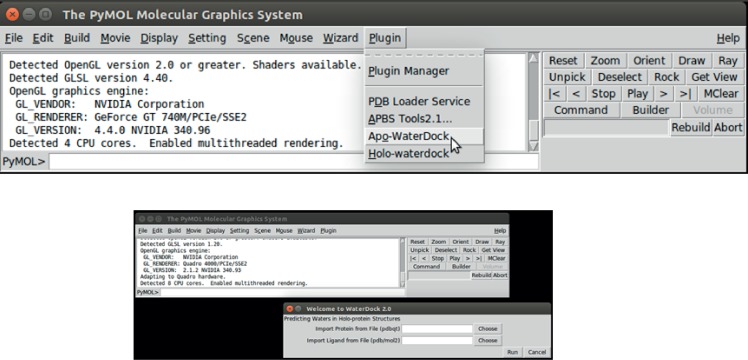
Snapshots of the PyMOL plugins developed for WaterDock pipelines.

To make WaterDock 2.0 easy to use, we developed a graphical user inter-face (GUI) based on PyMOL[43]. The GUI allows easy file loading and parameter specification for the protocol. Snapshots of the plugin installed within PyMOL are shown in **Fig 7**. The ‘*Apo*-WaterDock’ option is a PyMOL implementation of the original WaterDock previously scripted in R. The ‘*Holo*-WaterDock’ option is the WaterDock 2.0 pipeline developed here. The plugins along with a description of the installation/usage procedures and the necessary libraries are currently available at *https://github.com/bigginlab/WaterDock_pymol.git*. In addition, a command line version of WaterDock 2.0 is available *at https://github.com/bigginlab/WaterDock-2.0.git*.

## Supporting information

S1 Fig Functional group solvation. (PDF)

S2 Fig Analysis of the improvement in false-positive rate by WaterDock2.0 compared to the original WaterDock. (PDF)
