# Supplementary material for: Waterdock 2.0: Water placement prediction for Holo-structures with a pymol plugin
Source: PLoS One. 2017 Feb 24;12(2):e0172743. doi: 10.1371/journal.pone.0172743 (PMC5325533; doi:10.1371/journal.pone.0172743)

S1 Figure

(**A**) Schematic illustration of the imaginary X, Y and Z axes placed around the functional groups. The angle made by the ligand atom – O_{water} vector with the X and Z axes are ϕ and θ respectively. (**B-F**) The distribution of water-sites around five polar functional groups plotted as a 2D histogram of the two angles ϕ and θ. **(B)** Carbonyl **(C)** Carboxyl **(D)** Ether **(E)** Phosphoryl **(F)** Imine.

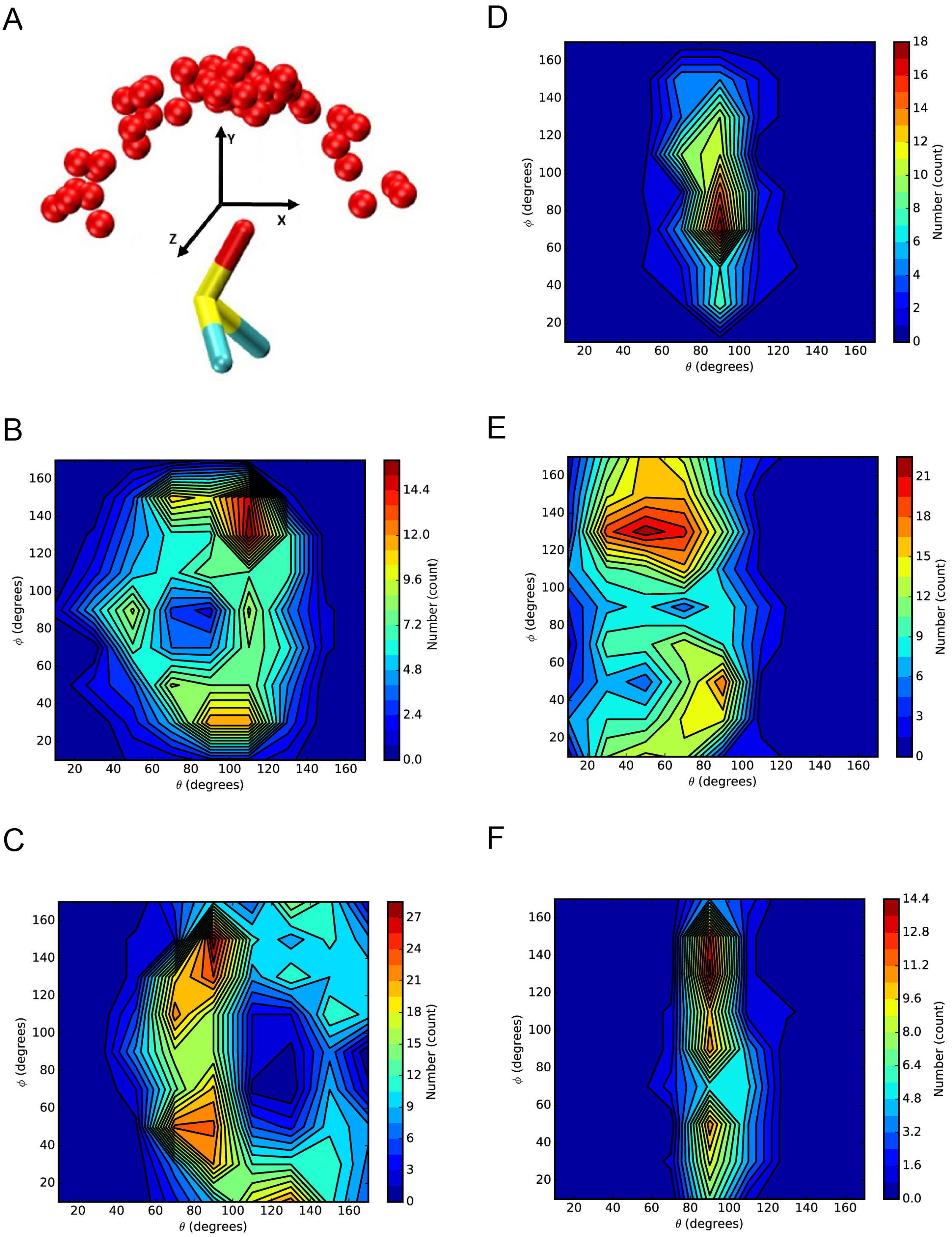

Supplement: S1 Fig — (PDF) [file pone.0172743.s001.pdf]
