# Supplementary material for: Waterdock 2.0: Water placement prediction for Holo-structures with a pymol plugin
Source: PLoS One. 2017 Feb 24;12(2):e0172743. doi: 10.1371/journal.pone.0172743 (PMC5325533; doi:10.1371/journal.pone.0172743)
Supplement: S2 Fig — (PDF) [file pone.0172743.s002.pdf]

S2 Figure

Comparison of the hit ratio (χ)[a], miss ratio (μ)[b] and reliability factor (ρ)[c] of the original and the newer WaterDock protocol as appplied to the common dataset of 14 OppA crystal structures based on the approach proposed by [1]. The average values are also shown as dotted lines. The original and new protocols could predict 95 and 92 waters respectively. The largest difference is evident in the miss-ratio plot of μ with the newer protocol having markedly lower number of false-positive predictions. Put together, the newer protocol has a better reliability ratio (ρ) of 0.82 compared to 0.79 of the original protocol.

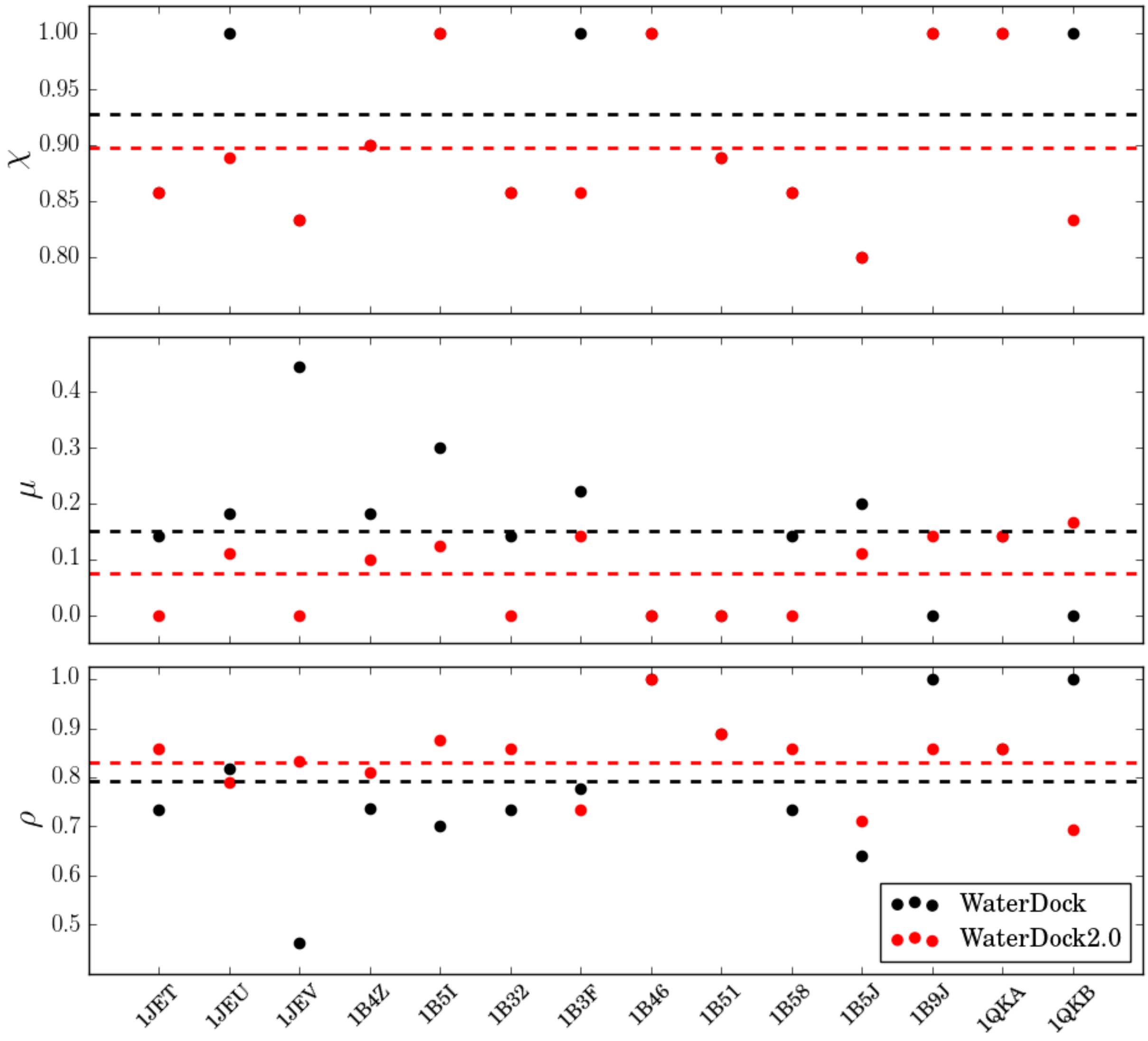

[a]The hit ratio χ measures the success of the program and is defined as:

$$\chi = \frac{N_{correct}}{N_{crystal}}$$

where $N_{crystal}$ is the number of crystallographic waters and $N_{correct}$ is the number of them correctly predicted (within 2.0 Å). Thus, χ is 0 if none of the crystallographic waters are predicted and 1 if all are predicted.

[b]The false-positive predictions are measured using the *miss-ratio* μ defined as:

$$\boldsymbol{\mu} = \frac{\boldsymbol{N_{false}}}{\boldsymbol{N_{total}}}$$

where N_{false} is the number of sites predicted without crystallographic waters within 2.0 Å. N_{total} is the total number of predictions and is the sum of $N_{correct}$ and N_{false}. Thus, μ is zero if there exist no false-positive predictions and 1 when all the placed water molecules are false-positives.

[c]The total reliability of the protocol is measured using a reliability parameter ρ defined as the product of χ and (1-μ):

$$\boldsymbol{\rho} = \boldsymbol{\chi}(\mathbf{1} - \boldsymbol{\mu})$$

Thus, ρ ranges from 0 to 1 based on the accuracy of the protocol.

References

1. Morozenko A, Stuchebrukhov AA (2016) Dowser++, a new method of hydrating protein structures. Proteins: Struct Func Bioinf 84: 1347-1357.
